# Maternal RSV Vaccination and Complementary Infant-Protection Strategies in Singapore: A Modified Nominal Group Technique Consensus Study

**DOI:** 10.3390/pathogens15101094

**Published:** 2026-10-20

**Authors:** Chia Yin Chong, Anne Goh, Anita Sugam Kale, Julian Hean Leng Kang, Bee Wah Lee, Manisha Mathur, Shephali Tagore

**Affiliations:** 1 Infectious Diseases Service, Department of Paediatrics, KK Women’s and Children’s Hospital, Singapore 229899, Singapore; chong.chia.yin@singhealth.com.sg; 2 Duke-NUS Medical School, Singapore 169857, Singapore; 3 Yong Loo Lin School of Medicine, National University of Singapore, Singapore117597, Singapore; 4 Lee Kong Chian School of Medicine, Nanyang Technological University, Singapore 308232, Singapore; 5 Paediatric Allergy Service and Respiratory Medicine Service, Department of Paediatrics, KK Women’s and Children’s Hospital, Singapore 229899, Singapore; anne.goh.e.n@singhealth.com.sg; 6 Department of Obstetrics and Gynaecology, Division of Maternal-Fetal Medicine, National University Hospital, Singapore 119074, Singapore; anita_sugam_kale@nuhs.edu.sg; 7 NUS Yong Loo Lin School of Medicine, Singapore 117597, Singapore; 8 Department of Obstetrics and Gynaecology, KK Women’s and Children’s Hospital, Singapore 229899, Singapore; julian.kang.h.l@singhealth.com.sg (J.H.L.K.); manisha.mathur@singhealth.com.sg (M.M.); 9 Department of Paediatrics, Yong Loo Lin School of Medicine, National University of Singapore, 1E Kent Ridge Road, NUHS Tower Block, Level 12, Singapore 119228, Singapore; paeleebw@nus.edu.sg; 10 Department of Maternal Fetal Medicine, KK Women’s and Children’s Hospital, Singapore 229899, Singapore

**Keywords:** respiratory syncytial virus, maternal immunisation, RSVpreF, nirsevimab, consensus, nominal group technique, Singapore

## Abstract

Background: Respiratory syncytial virus (RSV) is a leading cause of lower respiratory tract disease in infants and a major contributor to infant morbidity and mortality. A previous Singapore consensus addressed RSV disease burden and the role of the monoclonal antibody, nirsevimab, but did not extend to maternal RSV vaccination or its complementary use with long-acting monoclonal antibodies (mAbs). We sought structured expert consensus on positioning maternal RSV vaccination and a complementary prevention strategy in Singapore. Methods: A modified nominal group technique (mNGT) was used. A multidisciplinary panel of seven Singapore-based experts in obstetrics, including maternal–fetal medicine and paediatrics (respiratory, infectious disease, immunisation, and immunology), reviewed a pre-circulated targeted evidence review and four draft statements across four themes (burden, preventive pathways, maternal RSV vaccination, and complementary strategy), with the opportunity to submit written comments in advance. At a live virtual meeting on 16 June 2026, facilitated by a medical communications agency, the panel first discussed the evidence underpinning each draft statement and then voted openly (non-anonymously) on each statement in sequence. Consensus was predefined as ≥70% agreement. Statements that did not reach consensus were refined collectively, with each expert contributing in turn, and re-voted. Results: All seven experts attended and voted. Two statements reached consensus in Round 1 (Theme 2, 7/7; Theme 4, 6/7) and two after a single round of modification (Theme 1, 0/7 and then 7/7 after “first months of life” was specified as “first six months of life”; Theme 3, 3/7 and then 6/7 after “eligible pregnancies” was explicitly defined). The final statements cover RSV burden in the first six months of life, informed choice of preventive pathway, maternal RSV vaccination as an antenatal option for eligible pregnancies, and a complementary strategy in which a long-acting mAb (nirsevimab) is considered for infants not adequately protected by maternal vaccination. Conclusions: This expert consensus supports maternal RSV vaccination as an antenatal prevention option for eligible pregnancies in Singapore and a complementary strategy with nirsevimab for infants not adequately covered by maternal vaccination. The statements represent expert consensus intended to inform, not replace, national guidance.

## 1. Introduction

Respiratory syncytial virus (RSV) is the leading cause of acute lower respiratory tract infection (LRTI) and hospitalisation in infants worldwide. The burden of RSV LRTI is maximally concentrated in the first few months of life globally [1]. Young infants are particularly vulnerable because they are at greatest risk of severe bronchiolitis and pneumonia and have limited capacity to mount durable protective immunity [1,2]. In Singapore, RSV is the single most common cause of paediatric hospitalisation for viral respiratory infection, accounting for 42% of laboratory-confirmed viral respiratory detections between 2011 and 2016 [3]. It circulates throughout the year, with only a modest seasonal increase between May and September [2,3,4]. The burden falls hardest in the first months of life, where RSV is responsible for an estimated 33.5 hospitalisations per 1000 child-years among infants under 6 months of age [2], and it also accounts for 47% of all bronchiolitis and pneumonia admissions in that age group [2].

Singapore’s Health Sciences Authority (HSA) has registered three immunoprophylactic agents to prevent RSV disease in infants: the maternal RSV prefusion F (RSVpreF) vaccine [5] and two long-acting monoclonal antibodies (mAbs), nirsevimab [6] and clesrovimab [7]. Three distinct concepts warrant differentiation in this context: regulatory approval (HSA registration, which defines the licensed indication and the gestational-age or infant-age window), national recommendation (inclusion in a national immunisation schedule or formal national guidance), and implementation (routine availability, funding, and uptake within established care pathways). At the time of writing, all three products are approved by HSA in Singapore [5,6,7]. However, nirsevimab has been positioned as an option for infants in a prior Singapore expert consensus [4], which is not equivalent to national guidance, and none of the three products has yet been incorporated into a national immunisation schedule or a funded national programme. Maternal RSVpreF (Abrysvo) was approved by HSA in July 2024 for administration at 32 to 36 weeks of gestation [8], distinct from the 28–36 weeks used in several other jurisdictions, including the United Kingdom, where the product is both licensed and recommended from 28 weeks, and Australia, where it is registered from 24 weeks but routinely recommended from 28 weeks [9,10,11,12]; the Singapore window reflects the use of the United States Food and Drug Administration label (32–36 weeks) as the reference label for the HSA regulatory review [5,13]. It confers passive protection from birth through transplacental transfer of vaccine-induced neutralising antibodies [5], covering the period of greatest vulnerability [14]. Long-acting mAbs provide passive immunoprophylaxis when administered to the infant [4,6]. Nirsevimab is licensed in Singapore for neonates and infants entering or during their first RSV season and for children up to 24 months of age who remain vulnerable to severe RSV disease in their second season [6]. Clesrovimab, a more recently approved long-acting mAb, was subsequently registered in Singapore for infants entering their first RSV season [7]; however, its supporting evidence base and recommendations continue to evolve following the recent approval [7,15]. Reflecting the public-health importance of both approaches, the World Health Organisation (WHO) recommends that all countries introduce either maternal RSV vaccination or infant long-acting mAb to prevent severe RSV disease in young infants [15]. A prior structured Singapore consensus has addressed RSV disease burden and the role of long-acting mAbs; however, its stated objective was infant mAb prophylaxis rather than maternal RSV vaccination or how the two modalities could be used together as complementary infant-protection strategies [4].

Additionally, although both maternal RSV vaccination and infant long-acting mAbs are efficacious in preventing severe RSV disease in infants, they differ in timing, route of administration, and the circumstances under which each could best protect the infants [15]. In a growing number of jurisdictions, they are being deployed together rather than as mutually exclusive alternatives [9,10,11,12,16,17]. Several national programmes, including Australia’s 2025 hybrid RSV Maternal and Infant Protection Program, now combine antenatal maternal RSV vaccination between 28–36 weeks of gestation with infant mAb administration for infants not adequately covered by maternal vaccination [11,12]. Similarly, in the United Kingdom (UK), the Health Security Agency recommends maternal RSVpreF to every pregnant woman from week 28 of pregnancy, year-round, and at any time up to delivery [9,10]. Nirsevimab is reserved for infants not adequately protected by maternal vaccination, such as those of unvaccinated or severely immunosuppressed mothers, those born within 2 weeks of maternal vaccination, and other high-risk infants [9,10,11,12,16]. Such hybrid strategies aim to address residual protection gaps associated with either modality alone. In Singapore, antenatal care attendance is near-universal, established maternal immunisation programmes (influenza and pertussis) achieve moderate-to-high uptake [18,19], and RSVpreF is approved for use between 32 and 36 weeks of gestation [5]. However, maternal RSV vaccination is not yet the subject of national guidance in Singapore, and there is no structured local advice on how to position it within established antenatal pathways or how the two modalities may be used together to optimise infant protection.

Hence, this study aimed to (i) develop expert consensus statements on maternal RSV vaccination in Singapore and (ii) define how maternal vaccination and long-acting mAbs could be used as complementary infant-protection pathways. Four themes were specified a priori: (1) burden of RSV in infants, (2) infant RSV preventive pathways, (3) maternal RSV vaccination, and (4) complementary strategies.

## 2. Materials and Methods

This consensus study was designed and is reported in accordance with the ACcurate COnsensus Reporting Document (ACCORD) guideline for consensus methods in biomedical research [20].

### 2.1. Consensus Method and Rationale

A modified nominal group technique (mNGT) was used. The NGT is a structured consensus method that combines silent individual idea generation, round-robin contribution from every participant, structured group discussion, and voting; it is designed to give each participant an equal opportunity to contribute and to make the basis for decisions transparent [21,22]. The “modified” element in the present study was the addition of a pre-elicitation phase, in which an evidence review and draft statements were circulated before the meeting and written comments were invited, consistent with published adaptations of the NGT [23]. An mNGT was selected in preference to a Delphi process for three reasons. First, the objective was to refine a small number of statements in an area where the evidence base is evolving rapidly; the NGT permits real-time clarification of evidence, immediate iterative refinement of wording, and structured exchange across obstetric and paediatric disciplines, whereas a multi-round Delphi would have delayed completion by several months without a corresponding gain in rigour for four statements [21,22,24]. Second, the NGT is explicitly recommended for small, purposively selected expert groups, with published guidance suggesting that 5 to 12 participants is optimal for balancing diversity of perspective against the ability of every member to contribute meaningfully [21,22]. Third, the NGT generates a documented rationale for each modification, which we considered important for statements that could inform practice. The panel size of seven was determined prospectively as the number required to cover the pre-specified disciplines (paediatric infectious diseases and immunisation, paediatric respiratory medicine, paediatric allergy and immunology, general obstetrics and gynaecology, and maternal–fetal medicine), with at least one representative each and both major maternity providers represented, while remaining within the recommended NGT range. Several design features were used to reduce dominance effects, conformity pressure, and the influence of seniority during the live meeting: (i) the pre-elicitation phase allowed each expert to form and record a view before exposure to the group; (ii) discussion after any statement that failed to reach the consensus threshold followed the NGT round-robin format, in which every expert in turn stated their position and reasons before open discussion; (iii) the meeting was chaired by a facilitator from the medical communications agency who was independent of the panel (the facilitator was not a panel member, had no voting rights, and recorded but did not author changes to statement wording); and (iv) statements were voted on individually and in sequence, with the pre-specified threshold fixed in advance.

### 2.2. Preparatory Materials and Process

A medical communications agency (Transform Medical Communications, Auckland, New Zealand) prepared the pre-meeting materials, including a brief methodological note describing the mNGT process and voting rules, a targeted review of the scientific literature, and four draft statements, each explicitly linked to the supporting evidence. The agency was contracted by the funder to provide methodological and medical writing support (see Section 2.5).

#### 2.2.1. Evidence Identification

The evidence review was a targeted (non-systematic) review. Searches were conducted in PubMed/MEDLINE and Google (including Google Scholar), without date restriction, between April and June 2026, using combinations of the terms “respiratory syncytial virus” OR “RSV”; “infant” OR “neonate” OR “pregnan*” OR “maternal”; “RSVpreF” OR “maternal vaccin*” OR “maternal immuni*”; “nirsevimab” OR “monoclonal antibody*”; “Singapore”; and “effectiveness” OR “efficacy” OR “safety” OR “burden” OR “hospitali*”. Searches were supplemented by hand-searching (i) Singapore regulatory and formulary sources (Health Sciences Authority; National Drug Formulary) for the approved prescribing information of each product; (ii) national and international policy documents (WHO position paper; UK Health Security Agency Green Book and programme guidance; Australian Immunisation Handbook and Australian Technical Advisory Group on Immunisation resources); (iii) abstract books of the European Society for Paediatric Infectious Diseases (ESPID) 2025, European Society of Clinical Microbiology and Infectious Diseases (ESCMID) Global 2025 and RSV Vaccines for the World (RSVVW) 2026 congresses for programme-effectiveness data not yet published in full; and (iv) the reference lists of the prior Singapore RSV consensus [4] and included reviews. Eligibility criteria were English-language sources reporting original data or formal guidance on the epidemiology or burden of RSV in infants in Singapore or comparable high-income settings; the efficacy, effectiveness, safety, or immunogenicity of maternal RSVpreF vaccination or nirsevimab; or national policy and programmatic experience with maternal or infant RSV-prevention strategies. Within each domain, evidence was prioritised in the following order: Singapore-specific data; randomised controlled trials and their pre-specified analyses; large population-based effectiveness and safety studies; national programme evaluations; and, where no peer-reviewed source was available, congress abstracts and preprints, which were labelled as such in the evidence review. The evidence review was organised as a narrative summary with an evidence table for each of the four themes listing each source, its design, population, key results, and evidentiary status (peer-reviewed publication, congress abstract, preprint, or regulatory/policy document). The review encompassed maternal RSVpreF vaccination and nirsevimab only. Clesrovimab was approved in Singapore shortly before the consensus meeting; clesrovimab data were not included in the evidence review and were not discussed by the panel, and the consensus statements should not be read as applying to it. After the meeting, sources reviewed by the panel as preprints or congress abstracts were updated to their full publications. No evidence added after the meeting informed the votes.

#### 2.2.2. Development of Draft Statements

The medical communications agency proposed the four draft statements based on the evidence sought; each statement was cross-referenced to the specific evidence items in the review that supported it. One statement was drafted under each of the four pre-specified themes: (i) burden, (ii) preventive pathways, (iii) maternal RSV vaccination, and (iv) complementary strategy.

#### 2.2.3. Pre-Elicitation Phase

The pre-meeting materials were circulated to all seven experts 1 week before the meeting. Experts were asked to review the evidence, consider whether each draft statement was supported by and proportionate to the evidence presented, and return written comments if they wished. All experts returned written comments before the meeting; these were collated by the facilitator and presented at the start of the relevant discussion so that views formed independently were available to the group before any vote. Draft statements were not altered before the meeting on the basis of these comments; all modifications were made by the panel during the meeting.

### 2.3. Panel Composition

The expert panel comprised seven senior Singapore-based clinicians spanning the disciplines most relevant to maternal–infant RSV prevention: paediatric infectious diseases and immunisation, paediatric respiratory medicine, paediatric allergy and immunology, general obstetrics and gynaecology, and maternal–fetal medicine. Panellists were identified purposively against pre-specified eligibility criteria: (i) a practising specialist in Singapore in one of the target disciplines; (ii) at least 10 years of consultant-level experience; (iii) a record of peer-reviewed publication, guideline authorship, or advisory-committee membership relevant to RSV, maternal immunisation, or infant vaccination; and (iv) an affiliation with one of the public tertiary hospital groups managing deliveries and paediatric admissions in Singapore’s public sector. Candidates were identified by the authorship of Singapore infectious diseases and maternal immunisation publications, publicly available credentials, and national immunisation programme experience. The funder identified and nominated the panellists. A total of 10 experts were initially approached. Of these, seven agreed to participate, and three declined (two due to unavailability, while one did not provide a reason). Of the seven who agreed, the invitation was withdrawn for one expert after the hospital department recommended another expert to represent the department in this initiative. An invitation was then extended to the recommended candidate, who subsequently agreed to participate. Representation from public health, health economics, primary care, national immunisation authorities, and patient or parent groups was considered during planning but not included, for the following reasons: the questions posed were framed as clinical positioning questions to be addressed by the clinicians who counsel pregnant women and manage infants with RSV; the consensus was not intended to make programme-funding or scheduling decisions, which are the remit of national authorities; and health-economic evidence was not a pre-specified evidence domain.

### 2.4. Voting and Consensus Definition

The consensus meeting was held as a live virtual meeting on 16 June 2026 and was chaired by a facilitator from the medical communications agency, who had no voting rights. Only the seven panel members voted. The meeting proceeded in three stages. First, the panel discussed the pre-circulated evidence review as a whole, including the pre-elicitation comments. Second, the panel reviewed the four statements in sequence. For each statement, the facilitator displayed the exact wording in full and read it aloud, the panel discussed it, and each expert then cast a vote of Agree (A) or Disagree (D). Voting was open (non-anonymous): votes were cast in the presence of the other panel members and were recorded individually against each expert’s name on a spreadsheet by the facilitator. Anonymous voting was not used because the objective of the NGT discussion stage is for each expert to state and justify their position. Absent members were to be recorded as blank and excluded from the denominator; in the event, none of the seven experts was absent for any vote. Consensus was defined a priori as ≥70% agreement among votes cast (A + D). This threshold is widely used in healthcare consensus studies and is the most frequently reported threshold in methodological reviews of NGT and Delphi studies [21,22,24]; it is also consistent with the ≥70% cutoff used in the previous Singapore RSV consensus [4], supporting comparability. Third, statements that did not reach the threshold in Round 1 (R1) entered a structured modification step. Following the NGT round-robin format, each expert explained their view of the statement and any proposed wording changes; the facilitator displayed candidate revisions on screen, and the panel then collectively agreed on the revised wording. Wording modifications were therefore proposed by the panel members and captured, not authored, by the facilitator. Individual votes were recorded; the reasons underlying individual Disagree votes and the substance of the panel discussion leading to each modification are summarised in Section 3 and in the footnote to Table 1. The revised statement was displayed in full and voted on in Round 2 (R2) using the same open procedure and the same, unaltered threshold.

### 2.5. Safeguards for Independence

Safeguards implemented to preserve independence were (i) all evidence review and statement drafting was performed by the agency and reviewed by the panel experts before the live consensus voting meeting; (ii) voting was done exclusively by experts (authors of this study); (iii) the pre-specified consensus threshold could not be altered once the meeting began; and (iv) all authors had full access to the voting data and take responsibility for the content. The funder had no role in the search strategy, evidence selection, drafting of the statements, meeting discussion, voting, or data analysis. Its involvement was limited to a compliance review of the pre-meeting materials and the manuscript, confined to verifying the absence of off-label or promotional content.

### 2.6. Scope and Status of the Statements

The statements are expert consensus statements: they express the panel’s collective interpretation of the evidence regarding the preferred positioning of currently registered interventions in the Singapore context. They are neither an evidence-graded clinical practice guideline (no formal Grading of Recommendations Assessment, Development and Evaluation [GRADE] or equivalent appraisal was undertaken) nor a policy recommendation issued by, or on behalf of, a national immunisation authority. Theme 1 summarises epidemiological evidence; Theme 2 addresses principles of access and informed choice; Themes 3 and 4 express the panel’s opinion on the clinical positioning of the interventions and the practical circumstances in which each is likely to be most useful. The statements are intended to inform clinicians and to serve as input to, rather than a substitute for, formal national guideline and policy development. Where the Theme 2 and Theme 4 statements refer to long-acting monoclonal antibodies, this refers to nirsevimab, the only long-acting mAb for which evidence was reviewed by the panel; the statements do not extend to clesrovimab or to any other long-acting mAb.

## 3. Results

All seven invited experts attended the virtual meeting and voted on every statement.

### 3.1. Overview of Consensus Outcomes

Four draft statements were voted on across a maximum of two rounds. Two statements reached consensus in Round 1 (R1): Theme 2 (7/7 agree) and Theme 4 (6/7 agree, 1 disagree). Two statements did not reach the threshold in R1, were modified through the round-robin procedure, and reached consensus in Round 2 (R2): Theme 1 (R1 0/7; R2 7/7) and Theme 3 (R1 3/7; R2 6/7, 1 disagree). No statement remained unresolved. The verbatim wording of each statement as voted in each round, together with the full vote distribution, is given in Table 1 and described in Section 3.2, Section 3.3, Section 3.4 and Section 3.5.

### 3.2. Theme 1: Burden

R1 statement (“In Singapore, RSV circulation occurs year-round, and disease burden is greatest in early infancy, with the highest hospitalisation burden occurring in the first months of life”) did not reach consensus (0/7 agree; 7/7 disagree; 0.0%). The unanimous rejection did not reflect disagreement with the substance of the statement; the panel agreed that RSV circulates year-round and that burden is concentrated in early infancy. Rather, the discussion showed that none of the experts was willing to endorse an unspecified, arbitrary age band (“the first months of life”); the panel wished the statement to name the exact period that the evidence supports for three related reasons. First, the phrase is imprecise: it does not specify a number of months and could be read as anything from the neonatal period to the whole of the first year, which the panel considered unacceptable in a statement intended to guide the timing of prevention. Second, the phrase is not the unit in which the Singapore evidence is reported: the local hospitalisation-rate and admission-proportion data that the panel had reviewed are stratified for infants aged under 6 months (33.5 hospitalisations per 1000 child-years; 47% of bronchiolitis and pneumonia admissions) [2]; the panel wished the statement to map directly onto the evidence presented. Third, it was noted that an unspecified “first months” could be misinterpreted by readers as the first month of life alone, which would understate the period over which passive protection from maternal vaccination is clinically relevant. Following the round robin, the panel collectively agreed to replace “the first months of life” with “the first six months of life”; no other wording was changed.

R2 consensus statement (100% agreement, 7/7): “In Singapore, RSV circulation occurs year-round, and disease burden is greatest in early infancy, with the highest hospitalisation burden occurring in the first six months of life.”

### 3.3. Theme 2: Preventive Pathways

R1 consensus statement (100% agreement, 7/7): “Preventive strategies such as maternal vaccination and long-acting monoclonal antibodies are relevant considerations for protecting infants. Ensuring access and enabling informed choice are important considerations for mother–infant pairs in Singapore.”

### 3.4. Theme 3: Maternal RSV Vaccination

R1 statement (“Maternal RSV vaccination should be considered as an antenatal prevention option for eligible pregnancies in Singapore, particularly because it can provide passive infant protection from birth through the period of highest vulnerability”) did not reach consensus (3 agree, 4 disagree; 42.9%). The discussion centred on a single issue: the term “eligible pregnancies” was undefined in the statement, and without an explicit definition, the statement could be read as endorsing vaccination outside the HSA-approved 32–36-week window (e.g., from 28 weeks as in the UK and Australia [9,10,11,12]) or without regard to contraindications. Two options were discussed: rewriting the sentence to embed the gestational window and contraindications in the statement itself or retaining the statement unchanged and defining “eligible pregnancies” in an explicit footnote. The panel collectively chose the latter so that the statement would remain readable and would not require amendment if the licensed window changes. The only modification between rounds was therefore the addition of the footnote marker (*) and its definition: “Eligible pregnancies: pregnancies in which the vaccine can be administered at the HSA-approved gestational age in Singapore (32–36 weeks) and in which there is no contraindication to maternal RSV vaccine (a history of severe allergic reaction, e.g., anaphylaxis, to the vaccine or any of its components)” [5]. No other word of the statement was changed (Table 1).

R2 consensus statement (85.7% agreement, 6/7; 1 disagree): “Maternal RSV vaccination should be considered as an antenatal prevention option for eligible pregnancies* in Singapore, particularly because it can provide passive infant protection from birth through the period of highest vulnerability.”

The reason for the single R2 Disagree vote was that “eligible pregnancies” is tied to the current Singapore licence (32–36 weeks) and to the contraindications in the Singapore prescribing information, which are limited to a history of severe allergic reaction (e.g., anaphylaxis) to the vaccine or any of its components [5]; the definition would require review if the licensed window were amended.

### 3.5. Theme 4: Complementary Strategy

R1 consensus statement (85.7% agreement, 6/7): “In Singapore, a complementary RSV prevention strategy should be considered, whereby maternal RSV vaccination is discussed as an antenatal prevention option for eligible pregnancies, with long-acting monoclonal antibody protection considered for infants who are not adequately covered by maternal vaccination, such as those whose mothers were unvaccinated, those born within 14 days of maternal vaccination, or high-risk children beyond early infancy.”

Because the statement reached the pre-specified threshold in R1, no modification was made. The panel agreed to define the term “high-risk children beyond early infancy” in the accompanying footnote. The single R1 Disagree vote did not reflect disagreement with the substance of the statement; this expert considered that it should go further and specify how the complementary strategy would be delivered in practice, in particular that mothers should be made aware of the availability of mAbs where maternal vaccination has not been given or where coverage is expected to be inadequate. The panel supported this point and agreed to record it as a discussion rather than lengthening the statement. The three example categories in the statement were adopted from the evidence reviewed rather than newly defined by the panel: infants of unvaccinated mothers and infants born within 14 days of maternal vaccination follow the RSVpreF prescribing information and the UK and Australian programme criteria [5,9,10,11,12]; “high-risk children beyond early infancy” refers to children up to 24 months of age who remain vulnerable to severe RSV disease in their second RSV season, as defined in the Singapore prescribing information for nirsevimab and the prior Singapore consensus [4,6] (e.g., chronic lung disease of prematurity requiring ongoing treatment, haemodynamically significant congenital heart disease, or severe immunocompromise). The panel also noted, in the context of the evidence on RSV F-protein escape variants included in the review [25,26], that susceptibility to viral escape is mAb- and epitope-specific; the panel did not evaluate this issue in relation to any product other than nirsevimab. The long-acting mAb considered by the panel was nirsevimab; the statement does not extend to clesrovimab, for which no evidence was reviewed.

**Figure 1:**
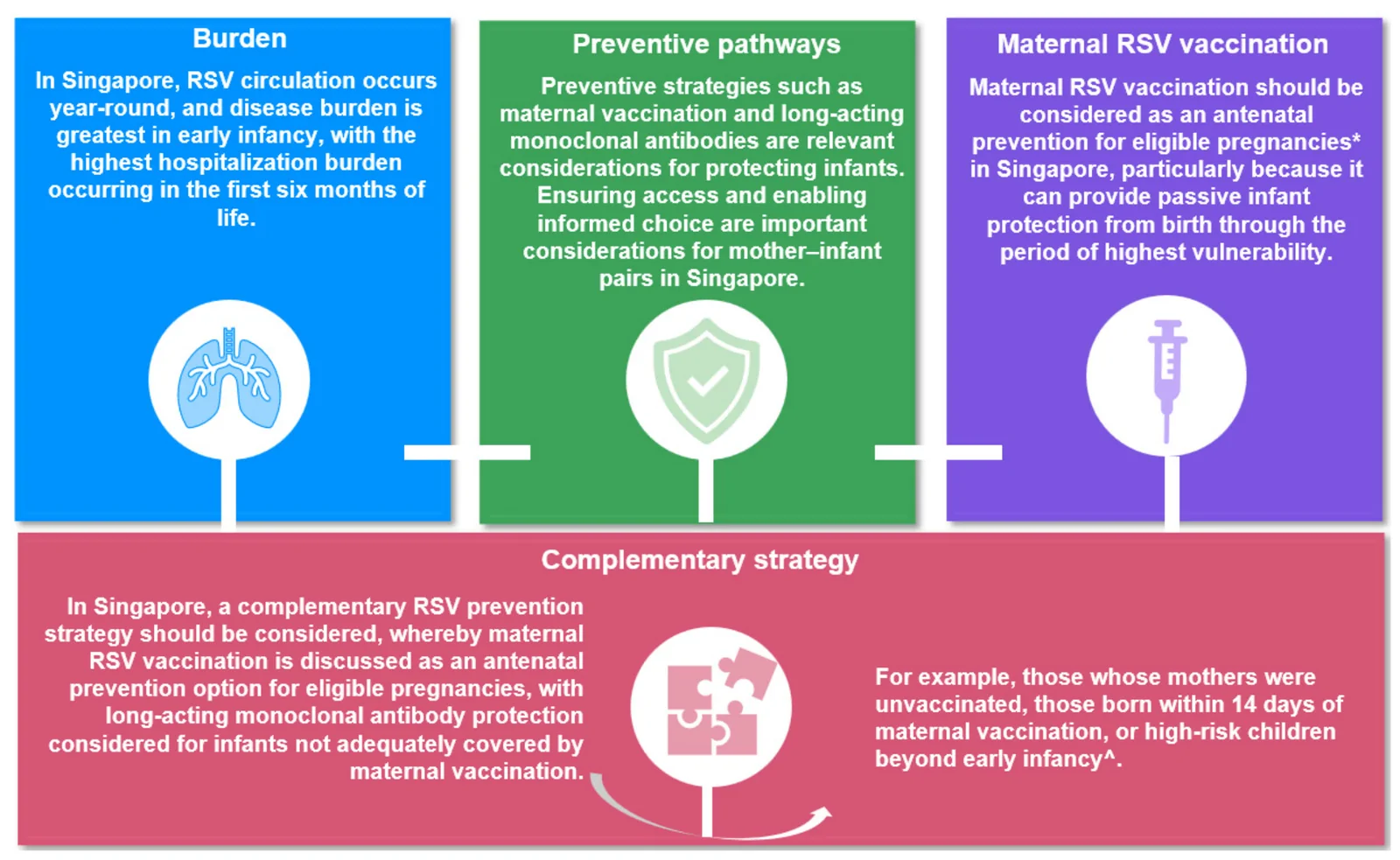
Summary of the four final expert consensus statements on RSV prevention in early infancy in Singapore. The figure summarises the statements agreed by this seven-member expert panel. The infant categories shown under the complementary strategy are illustrative examples taken verbatim from the Theme 4 statement and are defined in the footnotes below and to Table 1; they are not eligibility criteria. mAb, long-acting monoclonal antibody (nirsevimab); the statements do not extend to clesrovimab. * Eligible pregnancies: pregnancies in which the vaccine can be administered at the HSA-approved gestational age in Singapore (32–36 weeks) and in which there is no contraindication to maternal RSV vaccine (history of severe allergic reaction to the vaccine or any of its components) [5]. ^ High-risk children beyond early infancy: children up to 24 months of age who remain vulnerable to severe RSV disease in their second RSV season, as defined in the Singapore prescribing information for nirsevimab and the prior Singapore consensus [4,6]. Disclaimer: The statements summarised in this figure represent the consensus of a seven-member expert panel; they are not national guidance or a clinical practice guideline and are intended to inform, not replace, national guideline and policy development.

Figure 1 illustrates the final consensus recommendations on maternal RSV vaccination and complementary infant protection for Singapore.

## 4. Discussion

This mNGT consensus extends the prior Singapore RSV expert consensus [4] by addressing maternal RSV vaccination and articulating a complementary infant-protection strategy. The panel’s identification of peak burden in the first 6 months of life is grounded in the Singapore data reviewed by the panel. In Singapore, RSV circulates year-round with only a modest seasonal increase between May and September [3]. RSV accounts for an estimated 33.5 hospitalisations per 1000 child-years and approximately 3600 primary-care visits annually among children under 6 months of age [2]. It also represents almost half of all bronchiolitis and pneumonia admissions in that age group [2].

Among infants aged under 6 months, hospitalisation rates are approximately 2.5-fold higher than at 6 to 29 months [2]. This early concentration mirrors global modelling in which infants aged 1 to under 2 months account for the greatest share of RSV hospitalisations in high- and upper-middle-income settings (20.6% and 18.0%, respectively, versus a later peak of 15.5% at 2 to under 3 months in lower-income settings) [1]. Because maternal vaccination confers passive protection precisely through this period of highest vulnerability [14,27], aligning the prevention strategy with the earliest months of life is both biologically and epidemiologically coherent.

The unanimous endorsement of informed choice of pathway reflects both the availability of two efficacious modalities and evidence of parental preference. The World Health Organisation recommends that all countries introduce interventions to prevent severe RSV disease in young infants [15]. The role of nirsevimab within our recommendation framework draws on the prior Singapore consensus [4]. However, parental-preference data also reinforce a choice-of-pathway approach. Pregnant and postpartum women from a survey in Canada expressed a relative preference for vaccination during pregnancy over infant monoclonal antibody [28]. A European survey reported high acceptance of RSV immunisation, with many women favouring maternal vaccination [29]. However, real-world coverage data from the first United States season in which both modalities were available illustrate that healthcare provider recommendation is a key driver of uptake [30]. Provider recommendation was associated with higher uptake of both maternal immunisation and infant nirsevimab, whereas its absence was a leading reason for non-receipt of either modality [30].

The Theme 3 statement is an expert judgment; the evidence that the panel weighed in reaching it, and its limits, are set out here. The pivotal efficacy evidence, reviewed by the panel, comes from the MATISSE phase 3 trial conducted across 18 countries, in which bivalent RSVpreF administered during pregnancy reduced severe medically attended RSV-associated LRTI in infants by 81.8% within 90 days and by 69.4% within 180 days of birth [14,31]. It also reduced RSV-associated hospitalisation by 67.7% and 56.8% over the same intervals [31]. The maternal safety profile and infant birth and developmental outcomes were broadly comparable to placebo, a numerical imbalance in preterm births (5.7% vs. 4.7%) that was not statistically significant and was concentrated in one upper-middle-income country (South Africa) [14,31,32]. Subsequent real-world studies, several of which were reviewed by the panel, have been consistent with the trial findings on effectiveness [33,34,35,36] and maternal safety [37], although their designs differ (test-negative case control [33,34,35], cohort [33,36,37]), and one, the UK Health Security Agency cohort analysis, had been presented only as a conference poster and had not yet been peer-reviewed at the time of writing [36]. The largest, a UK Health Security Agency cohort analysis of 289,399 infants (approximately 90% of England births in 2024–2025), among whom 4594 were hospitalised for RSV, reported vaccine effectiveness of approximately 81% when mothers were vaccinated at least 2 weeks before birth, approximately 85% when vaccinated at least 4 weeks before delivery, and 69.4% in preterm infants; this analysis has so far been presented as a conference poster and has not yet undergone peer review [36]. In that cohort, infants of unvaccinated mothers comprised 55% of births but 87.2% of RSV hospitalisations [36]. UK programme monitoring also indicates that effectiveness increased with greater time from vaccination to birth, consistent with the kinetics of transplacental antibody transfer, while protection remained demonstrable when vaccination occurred as little as 10 days before delivery; no safety signals for preterm birth or Guillain-Barré syndrome emerged in these preliminary programme data at conference presentations [36,38]. Post-marketing safety observations from other countries have so far been consistent with this. In a real-world study in Argentina’s Córdoba Province, there was a 44% lower rate of preterm births and a 42% lower risk of low birth weight in the vaccinated cohort compared with the unvaccinated cohort [39]. However, the lower risk could be due to differences in healthcare-seeking behaviour and better follow-up in antenatal care [39]. Nevertheless, risk estimates for preterm birth and hypertensive disorders of pregnancy in the largest real-world data have clustered around the null elsewhere [36,38].

Health-economic evidence was included in the pre-elicitation evidence review circulated to the panel, but it was not a pre-specified evidence domain, and no draft statement or voting question addressed it; the analyses summarised in this and the following paragraph therefore did not form part of any consensus statement. They are nonetheless relevant to how the statements might be implemented in Singapore and are presented here as context. With that caveat, Singapore-specific modelling is consistent with maternal vaccination as an antenatal-prevention option. At 80% coverage, a year-round RSVpreF programme would prevent an estimated 308 infant hospitalisations and 1995 outpatient visits and reduce direct medical costs by S$2.15 million annually in Singapore while remaining cost-effective at vaccine prices up to S$237.68 per dose [40,41].

Singapore health-economic modelling data, which was presented to date as a conference poster and a peer-reviewed publication [40,41], is also consistent with a complementary strategy. A static cohort model compared nirsevimab alone with a complementary approach combining maternal RSVpreF with nirsevimab reserved for infants of unvaccinated mothers. The complementary approach achieved consistently greater reductions in RSV-related hospitalisations across the earliest months of life: 74% versus 47% at under 1 month, 73% versus 61% at 1 to under 2 months, and 87% versus 62% at 2 to under 3 months, relative to no intervention [40,41]. This advantage reflects the complementary roles of the two interventions: lower-cost maternal vaccination protects the majority of infants from birth. Nirsevimab, in turn, is reserved for the residual group: infants of unvaccinated mothers, those born too soon after vaccination. The complementary approach cost approximately S$149,000 per quality-adjusted life-year gained versus S$571,000 for nirsevimab alone. This is roughly four times more cost-effective for the same unit of health benefit [40,41]. These model outputs are sensitive to assumed vaccine price, coverage, and effectiveness and, being model-based, should be regarded as supportive context rather than as evidence of programme performance. The immunological plausibility of combining the two modalities in the same dyad is being examined in the PROMISE study, a phase 4 randomised trial of maternal RSVpreF, infant nirsevimab, or both [42]. In its interim analysis, bivalent RSVpreF increased maternal RSV-A and RSV-B neutralising titres 17.4- and 24-fold above baseline at delivery, and infant titres at birth were approximately 10- to 14-fold higher than those of infants of unvaccinated mothers (baseline approximately 1.1K IU/mL) [42]. The interim safety analysis showed no vaccine-related serious adverse events in either the mothers or the infants who received both interventions [42]. It should be emphasised that this is evidence of short-term safety of co-administration, not evidence of the absence of immunological interference: the interim report does not yet include the comparative infant neutralising-titre trajectories or clinical endpoints needed to determine whether pre-existing maternally derived antibody alters nirsevimab exposure or protection, or vice versa. Until those data are reported, the two modalities should be described as safe to layer within the dyad on current evidence, with the question of interference unresolved.

Real-world programmes that have adopted a layered approach provide early and, so far preliminary evidence of population-level impact; the Luxembourg and Australian data below were included in the review considered by the panel; at that time, both were available only as a congress abstract or preprint, and the Australian analysis has since been published in full [16]. In Luxembourg, a mixed strategy combining nirsevimab and maternal RSVpreF was associated with a 76.0% reduction in infant RSV hospitalisations in 2024–2025 relative to a season with no RSV immunisation, a further 44.5% reduction relative to the season in which nirsevimab was used alone, and an estimated 92.6% effectiveness against paediatric RSV hospitalisation (congress abstract; season-to-season comparisons are also subject to variation in RSV circulation) [17]. Australia’s 2025 hybrid RSV Maternal and Infant Protection Program (maternal vaccination from 28 weeks alongside nirsevimab for selected infants) shows that such a programme can be operationalised within an existing antenatal and immunisation system. Early impact and effectiveness data were generated through the Paediatric Active Enhanced Disease Surveillance–Influenza Complications Alert Network (PAEDS-FluCAN); they were shared with the panel and have since been published in full [16]. In a national test-negative case-control study of 3743 hospitalised children, effectiveness against RSV-associated hospitalisation in infants born after programme commencement was 82.6% (95% CI 70.6–89.7) overall, 81.5% (95% CI 68.4–89.2) for maternal vaccination, and 89.6% (95% CI 73.6–95.9) for nirsevimab, and RSV hospitalisations fell by 43.8%, 20.1%, and 8.5% in infants aged 0–<3, 3–<6, and 6–12 months, respectively, compared with the preceding year [16]. Both the United Kingdom and Australia have formalised this complementary structure in their national recommendations: maternal RSVpreF is offered in every pregnancy year-round (from 28 weeks of gestation), and nirsevimab is reserved for infants not adequately covered, namely those of unvaccinated or severely immunosuppressed mothers, those born within 2 weeks of maternal vaccination, and other high-risk infants [9,10,11,12,16]. These national frameworks are structurally similar to the strategy endorsed by the present panel and pair the same two products in high-income settings. Their experience should nonetheless be extrapolated to Singapore with caution, and it is useful to separate the components that are plausibly transferable from those that require local validation. Plausibly transferable are the structural logic of the strategy (maternal vaccination as the default antenatal offer with mAb for defined residual groups), the biological determinants of protection (the vaccination-to-delivery interval; waning of maternal antibody over approximately 6 months), the year-round delivery model already operated in the UK and Australia, and the residual-group definitions, which derive largely from product labelling rather than from local epidemiology. Components requiring local validation include (i) the gestational window because Singapore’s licence is 32–36 weeks, whereas the UK licence is 28–36 weeks, the Australian registration extends from 24 weeks, and both national programmes recommend vaccination from 28 weeks [9,10,11,12], which narrows the opportunity window in Singapore [43,44]; (ii) healthcare financing and access because both comparator programmes are publicly funded and free at the point of care, whereas maternal RSV vaccination in Singapore is currently not reimbursed, which is likely to affect uptake and equity; (iii) achievable coverage because UK and Australian maternal RSV uptake figures cannot be assumed for Singapore and no published local RSV-specific uptake data yet exist; and (iv) the effectiveness of a hybrid programme in a tropical country, which has not been measured anywhere. Local surveillance of uptake, timing, and effectiveness will therefore be essential if the strategy is adopted.

The two modalities also differ mechanistically in ways that are relevant to, though not the principal basis for, using them in a complementary rather than substitutive manner. The mechanistic evidence summarised below, other than the final phase 2b analysis [27], was included in the pre-elicitation evidence review circulated to the panel, but no draft statement or voting question addressed it, and it did not form part of any consensus statement; it is presented here because it is relevant to the rationale for a complementary approach. RSVpreF induces a broad polyclonal antibody response targeting multiple epitopes (including antibodies to the prefusion-specific antigenic sites Ø and V) that is theoretically more resilient to single-mutation viral escape than the single-epitope binding of a monoclonal antibody [45]. Consistent with this breadth, vaccine-induced sera retained neutralisation against 65 RSV isolates, more than 24 of them monoclonal antibody-resistant [46]. In the final analysis of the phase 2b maternal trial, published after the consensus meeting and not reviewed by the panel, neutralising titres in infants of vaccinated mothers remained higher than in infants of placebo recipients through the first 6 months of life (estimated half-life 39–44 days) [27] and remained above or comparable to the palivizumab-referenced protective threshold associated with at least 97.5% protection against RSV intensive-care admission [31,47]. Vaccine-induced antibody is also detectable in breast milk (congress abstract) [48], and the mother herself is protected, with vaccine effectiveness of 82.1% over the 12 months after vaccination (95% CI: 67.0–90.3) [49]. Nirsevimab, by contrast, provides infant-level protection, but because it binds a single epitope (site Ø), it is theoretically more vulnerable to viral escape through substitutions at that binding site [25]. The evidence on this point should be interpreted with care; it was not the subject of any statement or vote, and the panel did not evaluate it as a determinant of the strategy. In a large French multicentre study of nirsevimab breakthrough infections, F-protein substitutions associated with reduced nirsevimab susceptibility in vitro were rare in RSV-A (approximately 1%) but were detected in a minority of RSV-B breakthrough isolates, principally at position 208 (e.g., N208D) [26]. Surveillance from the second season of nationwide nirsevimab use in France, first presented at ESCMID Global 2026 and since published in full [50], showed an increase in the proportion of RSV-B breakthrough isolates carrying such substitutions, from approximately 8% (2 of 24) in 2023–2024 to 12.5% (23 of 184) in 2024–2025 [50]; no resistant virus was detected among nirsevimab-naïve infants, consistent with selection under prophylaxis rather than community spread [50]. Several caveats apply. The denominators are small and are drawn from breakthrough infections in immunised infants, not from unselected circulating virus, so these proportions overstate population prevalence; the detection of a genotypic substitution associated with reduced in vitro susceptibility may not be equivalent to demonstrated loss of clinical effectiveness, and nirsevimab effectiveness against RSV hospitalisation has remained high in the same seasons; and surveillance coverage is uneven, so trends may reflect sampling. In contrast, RSVpreF-induced polyclonal sera retained neutralising activity against resistant variants in vitro [25,46]. Potential resistance mutations, therefore, are a reason for continued genomic surveillance under any strategy that relies on a single-epitope mAb. For completeness, and as contextual information added by the authors after the consensus exercise, long-acting mAbs are not interchangeable in this respect: clesrovimab, which was not evaluated by the panel, binds a different epitope (site IV) and differs from nirsevimab in physicochemical and pharmacokinetic properties, including net charge (isoelectric point 8.52 versus ≤7.0), half-life (approximately 45 versus 69 days), and volume of distribution [51,52]; the escape-variant and effectiveness considerations discussed here for nirsevimab therefore cannot be assumed to apply to clesrovimab, and the panel’s statements should not be read as extending to it.

Additionally, successful maternal immunisation is fundamentally dependent on a strong antenatal care (ANC) system, which serves as the principal platform through which pregnant women are identified, counselled, and vaccinated [53]. This dependence is exemplified by maternal RSV vaccination because RSVpreF must be administered within a defined gestational-age window as per the country-specific approved license and national recommendations and should also allow sufficient time for transplacental antibody transfer [5]. Sufficient ANC visits and appropriately timed late-pregnancy contacts are therefore prerequisites for achieving high, correctly timed vaccine uptake, further supported by provider recommendations, documentation, and reminder systems [43,44,54,55]. Singapore has a strong foundation in several of these respects: antenatal care attendance is near-universal and structured [18,56], maternal pertussis vaccination has been implemented with demonstrated effectiveness [57], and surveyed pregnant women have expressed generally favourable attitudes toward maternal influenza and pertussis vaccination [19]. These features informed the panel’s confidence that the antenatal platform can deliver maternal RSV vaccination. However, a strong antenatal infrastructure does not by itself establish adequate RSV-specific awareness, acceptance, affordability, accessibility, and implementation barriers specific to each modality warrant discussion. For maternal RSV vaccination, four barriers stand out. First, cost: maternal RSV vaccination is currently not reimbursed in Singapore, which is likely to limit uptake and generate socioeconomic disparities, as has been observed for other self-funded maternal vaccines. Second, the same local survey that found favourable attitudes also documented substantial knowledge gaps and suboptimal actual uptake of influenza and pertussis vaccination in pregnancy, with absence of a provider recommendation and safety concerns being leading reasons for non-receipt [19]; RSV is a newer vaccine with a narrower window and can be expected to face at least similar hesitancy. Third, provider recommendation is the strongest single driver of uptake for both maternal RSV vaccination and infant nirsevimab [30], and Singapore obstetric providers will require education, counselling tools, and time within late-pregnancy visits to deliver it. Fourth, RSV-specific awareness among pregnant women in Singapore has not been measured. Infant long-acting mAbs face barriers of their own: a higher unit cost than maternal vaccination [40,41]; administration after birth, so that, depending on when the dose is given, there may be an interval during which the infant is unprotected, rather than protection being in place from birth as with maternal vaccination [6,14]; an already crowded infant immunisation schedule into which an additional injection must be integrated; and, as for maternal vaccination, dependence on parental awareness of RSV and on a provider recommendation [30]. Translation of this consensus into practice will therefore depend on addressing cost and access, provider training, patient information, and reminder and documentation systems and on monitoring uptake; these are not established by the present study.

## 5. Limitations

This consensus reflects the views of a small multidisciplinary panel of Singapore-based experts and should not be interpreted as a substitute for formal national guideline development. Several sources of potential bias inherent to the design should be weighed when calibrating the strength and generalisability of the statements. First, the evidence review was targeted rather than systematic: although the search strategy and inclusion criteria are reported, no formal risk-of-bias assessment or certainty grading was undertaken. Second, the panel was small (seven members), although within the range recommended for NGT; with seven voters, five Agree votes (5/7, 71.4%) would have met the ≥70% threshold, but in practice, every final statement was supported by at least six of the seven experts (6/7 or 7/7), and the full vote distributions and reasons for dissenting votes are reported in Table 1 and Section 3.2, Section 3.3, Section 3.4 and Section 3.5. Third, experts were nominated by the funder against pre-specified eligibility criteria (Section 2.3) and recruited purposively from two public hospital groups. This may introduce selection bias as the panel overrepresents tertiary hospital specialists and underrepresents primary care, private-sector obstetrics, national immunisation authorities, and, importantly, pregnant women and parents, whose perspectives on access, cost, and informed choice were not a part of this study. Fourth, voting was open and conducted in a live meeting; despite the pre-elicitation phase, round-robin format, and non-voting facilitation, conformity pressure, the influence of senior members, and a tendency to converge after discussion cannot be excluded. Fifth, although the statements were informed by the available evidence, some data cited derive from emerging real-world studies, programme evaluations, and congress abstracts, a few of which had not undergone peer review at the time of writing; further evidence may refine current understanding of effectiveness, safety, implementation, and escape variants. Sixth, several cited effectiveness and safety studies were conducted outside Singapore in settings with seasonal RSV circulation, publicly funded programmes, and a wider gestational window, and the local cost-effectiveness analysis is model-based; these factors may limit the direct applicability of the underlying data to routine local practice. Finally, the exercise did not address programme design questions such as funding, procurement, scheduling, or the second-season use of mAbs, which fall within the remit of national authorities.

## 6. Conclusions

The Singapore expert panel concluded that RSV burden is greatest in the first 6 months of life. Maternal RSV vaccination was endorsed as an antenatal prevention option for eligible pregnancies (32–36-week gestation, no contraindication), and a complementary strategy in which nirsevimab is considered for infants not adequately protected by maternal vaccination was also supported. These are expert consensus statements from a small panel, reached by open voting on a targeted evidence review; they are intended to inform clinicians and to serve as input to national guideline and policy development, not to replace them, and their translation into practice will depend on local decisions on funding, access, and provider engagement and on prospective monitoring of uptake and effectiveness.

## Figures and Tables

**Table 1:** Expert panel voting results for proposed and final consensus statements across mNGT rounds.

Theme	Round	Consensus Statement	Agreement	Result
1. Burden	R1	In Singapore, RSV circulation occurs year-round, and disease burden is greatest in early infancy, with the highest hospitalisation burden occurring in the first months of life.	0% (0/7)	Modify and revote
	R2	In Singapore, RSV circulation occurs year-round, and disease burden is greatest in early infancy, with the highest hospitalisation burden occurring in the first six months of life.	100% (7/7)	Consensus
2. Preventive pathways	R1	Preventive strategies such as maternal vaccination and long-acting monoclonal antibodies are relevant considerations for protecting infants. Ensuring access and enabling informed choice are important considerations for mother–infant pairs in Singapore.	100% (7/7)	Consensus
3. Maternal RSV vaccination	R1	Maternal RSV vaccination should be considered as an antenatal prevention option for eligible pregnancies in Singapore, particularly because it can provide passive infant protection from birth through the period of highest vulnerability.	42.9% (3/7)	Modify and revote
	R2	Maternal RSV vaccination should be considered as an antenatal prevention option for eligible pregnancies * in Singapore, particularly because it can provide passive infant protection from birth through the period of highest vulnerability.	85.7% (6/7)	Consensus
4. Complementary strategy	R1	In Singapore, a complementary RSV prevention strategy should be considered, whereby maternal RSV vaccination is discussed as an antenatal prevention option for eligible pregnancies, with long-acting monoclonal antibody protection considered for infants who are not adequately covered by maternal vaccination, such as those whose mothers were unvaccinated, those born within 14 days of maternal vaccination, or high-risk children beyond early infancy.	85.7% (6/7)	Consensus

mNGT, modified nominal group technique; R1/R2, voting Round 1/Round 2. Consensus threshold: ≥70% agreement of votes cast (Agree + Disagree); panel of seven experts, all present and voting openly (non-anonymously) in both rounds; the facilitator did not vote. Agreement is shown as % (number agreeing/number voting); the remainder in each case voted Disagree (no abstentions). Themes 2 and 4 reached consensus as proposed in R1; Themes 1 and 3 were modified by the panel and re-voted, reaching consensus in R2. Wording changes between rounds are underlined: Theme 1, “first months of life” → “first six months of life”; Theme 3, addition of the footnote marker (*) defining “eligible pregnancies”; no other words were changed. Reasons for the single Disagree vote on each adopted statement: Theme 3 (R2), the dissenting expert considered that tying “eligible pregnancies” to the current licensed window (32–36 weeks) and to the contraindications in the Singapore prescribing information would require the definition to be reviewed if the licence were amended; Theme 4 (R1), the dissenting expert, supported the statement but considered that it should additionally specify that mothers be made aware of the availability of mAbs where maternal vaccination has not been given or coverage is expected to be inadequate (see Section 3.5). Basis for the modifications (panel discussion): Theme 1, the panel was not willing to leave the age band unspecified (“the first months of life”) and, after discussing the Singapore evidence presented, agreed that the first 6 months of life is the period that the evidence identifies as carrying the highest hospitalisation burden; Theme 3, the panel wanted clarity on exactly what constitutes an “eligible pregnancy” and agreed to define it in a footnote by reference to the HSA-approved gestational window and contraindications. * Eligible pregnancies: pregnancies in which the vaccine can be administered at the HSA-approved gestational age in Singapore (32–36 weeks) and in which there is no contraindication to maternal RSV vaccine (per the Singapore prescribing information, a history of severe allergic reaction, e.g., anaphylaxis, to the vaccine or any of its components) [5]. “High-risk children beyond early infancy” (Theme 4) refers to children up to 24 months of age who remain vulnerable to severe RSV disease in their second RSV season as defined in the Singapore prescribing information for nirsevimab and the prior Singapore consensus (e.g., chronic lung disease of prematurity requiring ongoing treatment, haemodynamically significant congenital heart disease, or severe immunocompromise) [4,6]; the panel did not define a new risk list. “Long-acting monoclonal antibodies” (Themes 2 and 4) refers to nirsevimab, the only long-acting mAb for which evidence was reviewed by the panel; the statements do not extend to clesrovimab [7].

## Data Availability

The draft and final statements and all voting data are reported in this article (Table 1 and Section 3.1, Section 3.2, Section 3.3, Section 3.4 and Section 3.5); the structure and sources of the evidence review are described in Section 2.2, Section 3.1, Section 3.2, Section 3.3, Section 3.4, Section 3.5 and Section 4 and cited in the reference list.
